# Assessing the Quality of ChatGPT Responses to Dementia Caregivers’ Questions: Qualitative Analysis

**DOI:** 10.2196/53019

**Published:** 2024-05-06

**Authors:** Alyssa Aguirre, Robin Hilsabeck, Tawny Smith, Bo Xie, Daqing He, Zhendong Wang, Ning Zou

**Affiliations:** 1 Department of Neurology, The University of Texas at Austin, Austin, TX, United States; 2 Steve Hicks School of Social Work, The University of Texas at Austin, Austin, TX, United States; 3 Glenn Biggs Institute for Alzheimer's & Neurodegenerative Diseases, Department of Neurology, University of Texas Health Science Center at San Antonio, San Antonio, TX, United States; 4 Department of Psychiatry and Behavioral Sciences, The University of Texas at Austin, Austin, TX, United States; 5 School of Information, The University of Texas at Austin, Austin, TX, United States; 6 School of Nursing, The University of Texas at Austin, Austin, TX, United States; 7 School of Computing and Information, University of Pittsburgh, Pittsburgh, PA, United States

**Keywords:** Alzheimer’s disease, information technology, social media, neurology, dementia, Alzheimer disease, caregiver, ChatGPT

## Abstract

**Background:**

Artificial intelligence (AI) such as ChatGPT by OpenAI holds great promise to improve the quality of life of patients with dementia and their caregivers by providing high-quality responses to their questions about typical dementia behaviors. So far, however, evidence on the quality of such ChatGPT responses is limited. A few recent publications have investigated the quality of ChatGPT responses in other health conditions. Our study is the first to assess ChatGPT using real-world questions asked by dementia caregivers themselves.

**Objectives:**

This pilot study examines the potential of ChatGPT-3.5 to provide high-quality information that may enhance dementia care and patient-caregiver education.

**Methods:**

Our interprofessional team used a formal rating scale (scoring range: 0-5; the higher the score, the better the quality) to evaluate ChatGPT responses to real-world questions posed by dementia caregivers. We selected 60 posts by dementia caregivers from Reddit, a popular social media platform. These posts were verified by 3 interdisciplinary dementia clinicians as representing dementia caregivers’ desire for information in the areas of memory loss and confusion, aggression, and driving. Word count for posts in the memory loss and confusion category ranged from 71 to 531 (mean 218; median 188), aggression posts ranged from 58 to 602 words (mean 254; median 200), and driving posts ranged from 93 to 550 words (mean 272; median 276).

**Results:**

ChatGPT’s response quality scores ranged from 3 to 5. Of the 60 responses, 26 (43%) received 5 points, 21 (35%) received 4 points, and 13 (22%) received 3 points, suggesting high quality. ChatGPT obtained consistently high scores in synthesizing information to provide follow-up recommendations (n=58, 96%), with the lowest scores in the area of comprehensiveness (n=38, 63%).

**Conclusions:**

ChatGPT provided high-quality responses to complex questions posted by dementia caregivers, but it did have limitations. ChatGPT was unable to anticipate future problems that a human professional might recognize and address in a clinical encounter. At other times, ChatGPT recommended a strategy that the caregiver had already explicitly tried. This pilot study indicates the potential of AI to provide high-quality information to enhance dementia care and patient-caregiver education in tandem with information provided by licensed health care professionals. Evaluating the quality of responses is necessary to ensure that caregivers can make informed decisions. ChatGPT has the potential to transform health care practice by shaping how caregivers receive health information.

## Introduction

Older adults have responded to the COVID-19 pandemic by increasing their internet-enabled behaviors, which include expanding their medical care to the use of web-based platforms [1]. Indeed the internet has become the most common source of information among dementia caregivers [2], and with recent advances in artificial intelligence (AI), caregivers will increasingly use AI to obtain information about health [3,4]. ChatGPT by OpenAI [5], an innovative, dialogue-based large language model that responds to complex natural language inquiries, holds great promise to improve the quality of life of patients with dementia and their caregivers by providing high-quality responses to meet their needs for information [4]. On the other hand, several studies have highlighted the limitations of generative AI models in health care, citing the lack of trust and reliability as some of the primary challenges [6,7]. Although there have been studies on the quality of ChatGPT responses to common questions about heart disease [8], cirrhosis [9], and bariatric surgery [10], to our knowledge, no studies have examined the quality of ChatGPT responses to real-world questions posed by dementia caregivers. We have addressed this gap by examining the quality of ChatGPT-3.5 responses to complex questions posted by dementia caregivers on social media.

## Methods

### Overview

From January to May 2023, a total of 60 social media posts representing dementia caregivers’ needs for information in 3 areas (memory loss and confusion, aggression, and driving; 20 posts per area) were selected from Reddit, a popular social media platform. These topics were chosen because they are common clinical themes that are often complex and difficult to navigate with potential safety implications. Four seed posts were used in each area to discover the additional 16 posts. Posts were excluded if the poster’s main question did not fall into the 3 aforementioned areas as verified by dementia clinicians or if the poster declared they were “venting” and/or no specific question was asked. Posts that were unclear on whether the person had a dementia diagnosis were excluded to avoid assessing posts that were not clearly dementia related. Word count for posts in the memory loss and confusion category ranged from 71 to 531 (mean 218; median 188), aggression posts ranged from 58 to 602 (mean 254; median 200), and driving posts ranged from 93 to 550 (mean 272; median 276). Of the 60 posts, the caregiver described the person with dementia as their parent (n=34, 56%), grandparent (n=22, 36%), uncle (n=2, 3%), or spouse (n=1, 1.6%). One post did not report relationship. The gender of the person with dementia was described as female in 57% (n=34) of posts and as male in 42% (n=25) of posts. One post did not report gender.

Three clinicians, each having more than 15 years of experience with patients with dementia and their caregivers, but from diverse disciplines (pharmacy, neuropsychology, and social work), assessed ChatGPT responses to the 60 posts using an adapted rating scale based on Hurtz et al’s [11] levels of cognitive complexity pertaining to clinical decision-making (Table 1). Responses received 1 point for each of the following characteristics: *factuality*, *interpretation*, *application*, *synthesis*, and *comprehensiveness*, with a scoring range of 0-5 for each response, where higher scores indicate higher quality. Table S1 in Multimedia Appendix 1 presents examples of posts for each topic area, ChatGPT responses, and clinician ratings for each response category.

**Table 1.** Description of rating scale categories used to measure the quality of ChatGPT responses.

Characteristic	Description
Factuality	Response did not contain inaccurate or false information.
Interpretation	Response adequately interpreted the poster’s main need, correctly disregarded nonpriority details, and did not recommend strategies that the poster had already tried.
Application	Response suggested tangible actions (eg, educational information, a change the caregiver could make, and communication strategies such as validation and redirection).
Synthesis	Response contained follow-up recommendations as needed (referrals to help beyond the caregiver-patient dyad, such as support groups, health care professionals, or other community resources).
Comprehensiveness	Response had strong depth, breadth; response was thorough and complete.

Although the results reported in this paper were based on raters’ consensus scores, we acknowledge the potential benefits of expanding on ChatGPT responses that originally received different scores. Initially, 1 rater gave a point for comprehensiveness when the majority of suggestions they would provide clinically were conveyed in ChatGPT’s response, but another rater did not give the point if they felt it was missing anything at all. It was agreed upon during consensus that if the majority of recommendations were provided, ChatGPT responses would receive full credit for *comprehensiveness*.

### Ethical Considerations

This study was approved by the institutional review boards of The University of Texas at Austin (STUDY00003358) and the University of Pittsburgh (STUDY20020007).

## Results

ChatGPT responses in the memory loss and confusion category ranged from 89 to 276 words (mean 170; median 165), 91 to 372 words in the aggression category (mean 221; median 234), and 65 to 359 words in the driving category (mean 175; median 130). At least 2 clinicians agreed on the ratings for all ChatGPT responses, with any disagreements resolved by discussion. ChatGPT scores ranged from 3 to 5. Overall, of the 60 responses, 26 (43%) received 5 points, 21 (35%) received 4 points, and 13 (21.7%) received 3 points (Table 2), suggesting high quality. There were no responses that scored a 0, 1, or 2; there were no fabricated responses; and no responses were considered harmful to posters. ChatGPT received the lowest ratings in *comprehensiveness*, followed by *interpretation*, and the highest ratings in *synthesis*, with only 2 out of 60 posts failing to receive the point (Table 3).

**Table 2.** Rating scale results by topic.

Score	Memory loss and confusion (n*=*20), n (%)	Aggression (n*=*20), n (%)	Driving (n*=*20), n (%)	Total (N=60), n (%)
3	6 (30)	3 (15)	4 (20)	13 (22)
4	7 (35)	6 (30)	8 (40)	21 (35)
5	7 (35)	11 (55)	8 (40)	26 (43)

**Table 3.** Number of ChatGPT points for each topic.

Characteristic	Memory loss and confusion (n=20), n (%)	Aggression (n=20), n (%)	Driving (n=20), n (%)	Total (N=60), n (%)
Factuality	17 (85)	19 (95)	20 (100)	56 (93)
Interpretation	17 (85)	17 (85)	13 (65)	47 (78)
Application	20 (100)	17 (85)	17 (85)	54 (90)
Synthesis	18 (90)	20 (100)	20 (100)	58 (96)
Comprehensiveness	9 (45)	15 (75)	14 (70)	38 (63)

## Discussion

### Principal Findings

In this study, ChatGPT responses to complex, real-world questions posted by dementia caregivers were assessed by dementia clinicians using a clinical decision-making rating scale. ChatGPT was found to produce high-quality responses, suggesting the potential of online chatbots to be a useful source of health information for dementia caregivers. The majority of responses contained factual information (n=56, 93%), with 78% (n=47) of responses correctly interpreting the poster’s main need. The majority (n=54, 90%) of ChatGPT responses contained tangible actions the caregiver could apply to their situation. In only 2 instances, follow-up referrals were not suggested when reviewers felt recommendations were needed.

ChatGPT also had limitations, primarily in the areas of *interpretation* and *comprehensiveness*. In 22% (n=13) of posts, ChatGPT recommended strategies that posters had already explicitly tried, or missed subtleties that affected the accuracy of recommendations, such as failing to recognize that a person placed in a “home” meant a nursing home facility and not a traditional home. In another instance, ChatGPT recommended considering short-term hospitalization, but the poster already disclosed the person with dementia was currently hospitalized. In 37% (n=22) of posts, ChatGPT’s response did not include information that dementia clinicians felt was important or was unable to anticipate future problems that a human clinician might choose to address in response to the same post. For example, if ChatGPT recommended a driving test, it did not suggest what to do if the patient in question refused to take the driving test. The data suggest that ChatGPT has strengths in providing objectively correct information (*factuality*, *application*, and *synthesis*) but is less successful in contextualizing the information it provides (*interpretation* and *comprehensiveness*).

### Limitations

Study limitations included potential sample bias and small sample size. Very few posters in this study identified as a spousal caregiver (n=1, 1.6%) even though national studies report that 60% of dementia caregivers are a spouse or partner [12]. In selecting social media posts for inclusion, we included only those in which it was clear that the individual had a diagnosis of dementia. Historically, racial and ethnic minority groups are less likely to seek or receive a dementia diagnosis; thus, our sample may have been skewed for race and ethnicity. Posts were from one specific platform, which risked including caregivers with a certain level of technology access and literacy. This study did not evaluate differences in ChatGPT responses at multiple time points, so no conclusions can be made regarding reproducibility. Raters were aware that responses were generated by ChatGPT, which could have influenced stricter grading. Although our 5-point scale graded specific aspects of ChatGPT responses, it might have had a ceiling effect.

### Conclusions

This study contributes to the currently small but rapidly growing literature on AI’s potential to assist patient-caregiver education by providing high-quality information. Our study illustrates that ChatGPT-3.5 can provide high-quality responses to most questions in the areas of memory loss and confusion, aggression, and driving. Future research should examine family caregivers’ receptiveness to using ChatGPT, as well as the usefulness of the responses from the perspective of family caregivers. Validated rating scales to assess the quality of ChatGPT responses are still in progress; the field would benefit from a reliable, validated method to evaluate the quality of AI responses to health care questions. We encourage future studies to expand on our findings and investigate how ChatGPT might be used in tandem with information provided by licensed health care professionals.

## Supplementary material

10.2196/53019 Multimedia Appendix 1 Scoring of responses generated from ChatGPT.

## Abbreviations

AI: artificial intelligence
