# Supplementary material for: Assessing the Quality of ChatGPT Responses to Dementia Caregivers’ Questions: Qualitative Analysis
Source: JMIR Aging. 2024 May 6;7:e53019. doi: 10.2196/53019 (PMC11089887; doi:10.2196/53019)
Supplement: Multimedia Appendix 1 [file aging-v7-e53019-s001.docx]

**Table S1. Scoring of responses generated from ChatGPT**

| Reddit Post – Memory Loss and Confusion | ChatGPT Response | Factual | Interpretation | Application | Synthesis | Comprehensiveness |
| --- | --- | --- | --- | --- | --- | --- |
| My grandparent has vascular dementia and lives at home with one of her older children. I go over to help give company when I can, and I think the hardest part is making her feel at home. She constantly asks when we are leaving to go home, as she thinks she isn't in her house and wants to go back to what we assume is the house she grew up in with her parents in a different state.   How can I answer her questions to make her feel at home? She mostly asks when we are leaving, if she needs money or how to pack for the trip. Every day when it's not freezing outside and in some that it is she packs her car with random things like she is going somewhere or brings things up and down the stairs trying to get ready. We of course have the keys hidden but leave the car doors unlocked because she can get angry when HER car isn't available to her. I've made a sign for her bedroom door that says her name and plan on making a memory book with a picture of her in her house with some family so she can maybe make the association on her own. But nothing really seems to make her feel better and she gets more and more restless and hard to deal with by the day.  I usually try to answer that she is home and that her family lives with her, and try to point out things in the house that she knows are hers -paintings she has done, or take her to her room to give her a tour. Sometimes she realizes that it is her house but usually not and she often answers with "I didn't know that." This is how every conversation goes and I just wonder if anyone has any tips for how to answer or make her feel more comfortable without trying to force the information on her? | As an AI language model, I may not have the same level of empathy as humans, but I can suggest some tips that may help you deal with your grandparent's situation.  Firstly, it's important to understand that your grandparent's confusion and restlessness are symptoms of her dementia, and it's not something she can control. Therefore, it's essential to be patient and understanding with her.  One approach you can take is to validate her feelings and acknowledge her concerns. For example, when she asks when you are leaving, you can say something like, "I understand that you want to go home, but we are already at your home. This is where you live with your family."  You can also try to redirect her attention to something that she enjoys or is familiar with. For instance, you can ask her about her favorite memories or hobbies, or play some music that she likes.  Creating a memory book with pictures and stories from her past may also help her make connections and feel more at ease. You can also try to involve her in activities that she used to enjoy, such as cooking or gardening, to give her a sense of purpose and accomplishment.  Lastly, it's crucial to take care of yourself and seek support from other family members or professionals if needed. Caring for someone with dementia can be challenging, and it's essential to prioritize your own well-being as well. | 1 | 1 | 1 | 1 | 1 |
| Reddit Post – Aggression | ChatGPT Response | Factual | Interpretation | Application | Synthesis | Comprehensiveness |
| Hello, my grandpa who’s 82 has dementia and is at the point where he can not take care of himself including hygiene. Me and my mother have been acting as his caretakers for years now but it’s past the point of being too much for us to handle. He has extreme outbursts that are starting to get violent and we’re not able to work due to having to take care of him. Unfortunately we cannot get IHHS because of insurance. We’ve tried placing him in a home but he refuses to go. Does anyone have advice on what we can do? At this point our household is becoming dangerous for everyone in this situation :( | I'm sorry to hear about your situation. It sounds like you and your mother are doing the best you can, but it's becoming too much to handle. Have you considered reaching out to local support groups or organizations that specialize in dementia care? They may be able to provide resources and assistance for your grandpa's care. Additionally, you could try talking to his doctor or a social worker to see if they have any recommendations or can provide guidance on next steps. It's important to prioritize everyone's safety in this situation. | 1 | 1 | 0 | 1 | 0 |
| Reddit Post- Driving | ChatGPT Response | Factual | Interpretation | Application | Synthesis | Comprehensiveness |
| i (51m) am looking after my mom (83f) who was diagnosed w alzheimer's in 2018 after exhibiting symptoms starting in 2016. she shouldn't be driving (there's plenty of evidence that my siblings and i have witnessed first hand). the neurologist reported her to the dmv upon diagnosis, but she somehow passed the dmv driving test. her insurance was canceled for a few months (i guess dmv notifies the insurer?) and keeping her from driving was quite difficult at that time (her screaming profanities at me, etc.).fast forward to 2020. she's still driving once/week to her friends house about 20 miles away. it needs to stop, so i concoct a plan (that her friend agrees with) that her keys go missing, and we'll ease her into using uber/lyft for the 20 mile trip. she refuses to take the uber, but can't really explain why. this was three days ago. since then, she has called me an average of 35 times/day while i'm away at work either asking me where her keys are or saying there's an emergency and i need to come home immediately. she has no recollection that she's calling me that often. when i get home, she wants me to solve the problem, and is relentless (although i sometimes get a break because she forgets the problem exists). this is killing me. part of me wants to "find" the keys, so that she can continue driving with the hope that something bad does happen so that this misery that is alzheimer's will finally end. then part of me says, others must certainly have been in this situation before and gotten through it, so i can too.so tell me reddit, how do i get over this hurdle w her??? | As an AI language model, I cannot provide medical advice. However, I suggest seeking professional help from a doctor or a therapist who can guide you on how to handle your mother's situation. It is essential to prioritize her safety and the safety of others on the road. You can also seek legal advice on how to revoke her driver's license if necessary. Remember that Alzheimer's disease is a challenging condition, and it is okay to ask for help. | 1 | 1 | 0 | 1 | 0 |
